# Supplementary material for: Sampling techniques and genomic analysis of biological material from artworks
Source: J Forensic Sci. 2025 Feb 7;70(2):476–89. doi: 10.1111/1556-4029.15701 (PMC11874228; doi:10.1111/1556-4029.15701)
Supplement: Supplementary file 1 — Data S1: Supporting Information. [file JFO-70-476-s001.docx]

**SUPPLEMENTAL DATA**

**Sampling techniques and genomic analysis of biological material from artworks**


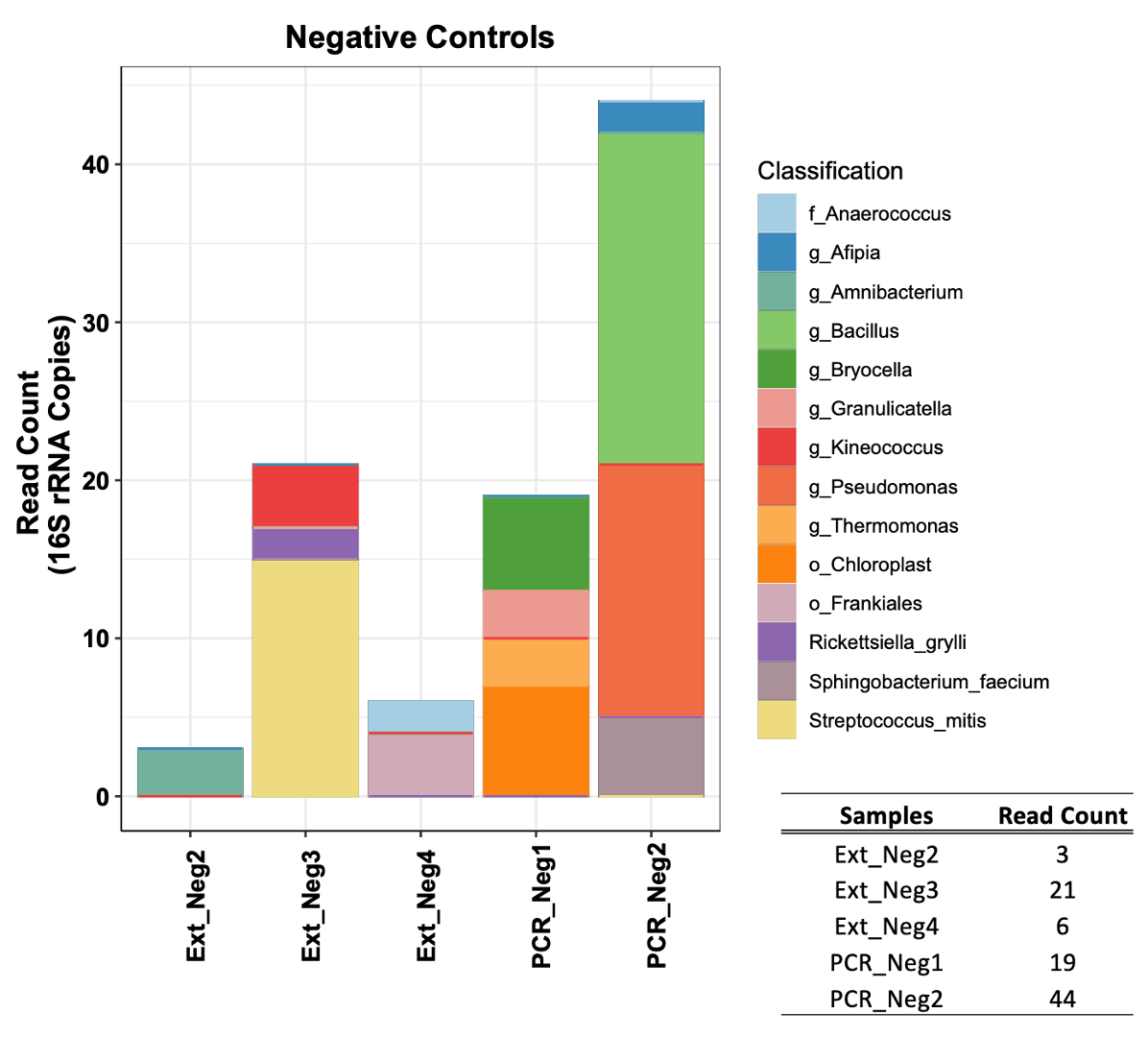


FIGURE S1 Low contamination was identified in the extraction and PCR negative control samples. Bacteria identified in the negative controls are associated with trace amounts of water- or reagent-associated bacteria taxa (e.g., *Pseudomonas* sp.) and human commensal bacteria (e.g., *Streptococcus mitis*).

**
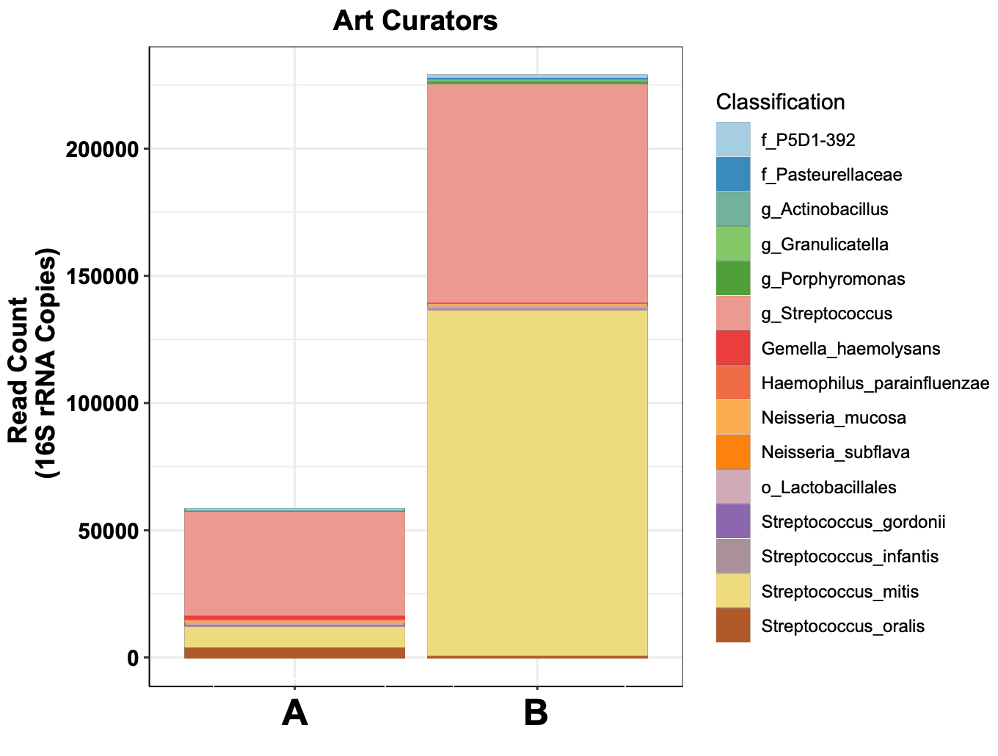
**

FIGURE S2 Buccal swab control samples from the art curators to monitor potential human contamination. The plot represents the total read count of the top 15 bacterial taxa identified in the samples. A total of 38 OTUs were detected in the art curator’s samples. Based on the full length 16S gene (V1-V9), a total of 58,821 reads were identified in sample **A** and 229,189 reads were identified in sample **B**. For both, host commensal bacteria *Streptococcus* sp. and *Streptococcus mitis* were most abundant. Human commensals *Streptococcus* and *Staphylococcus* sp. were detected in the centuries-old artwork samples (Figures S3-S4).


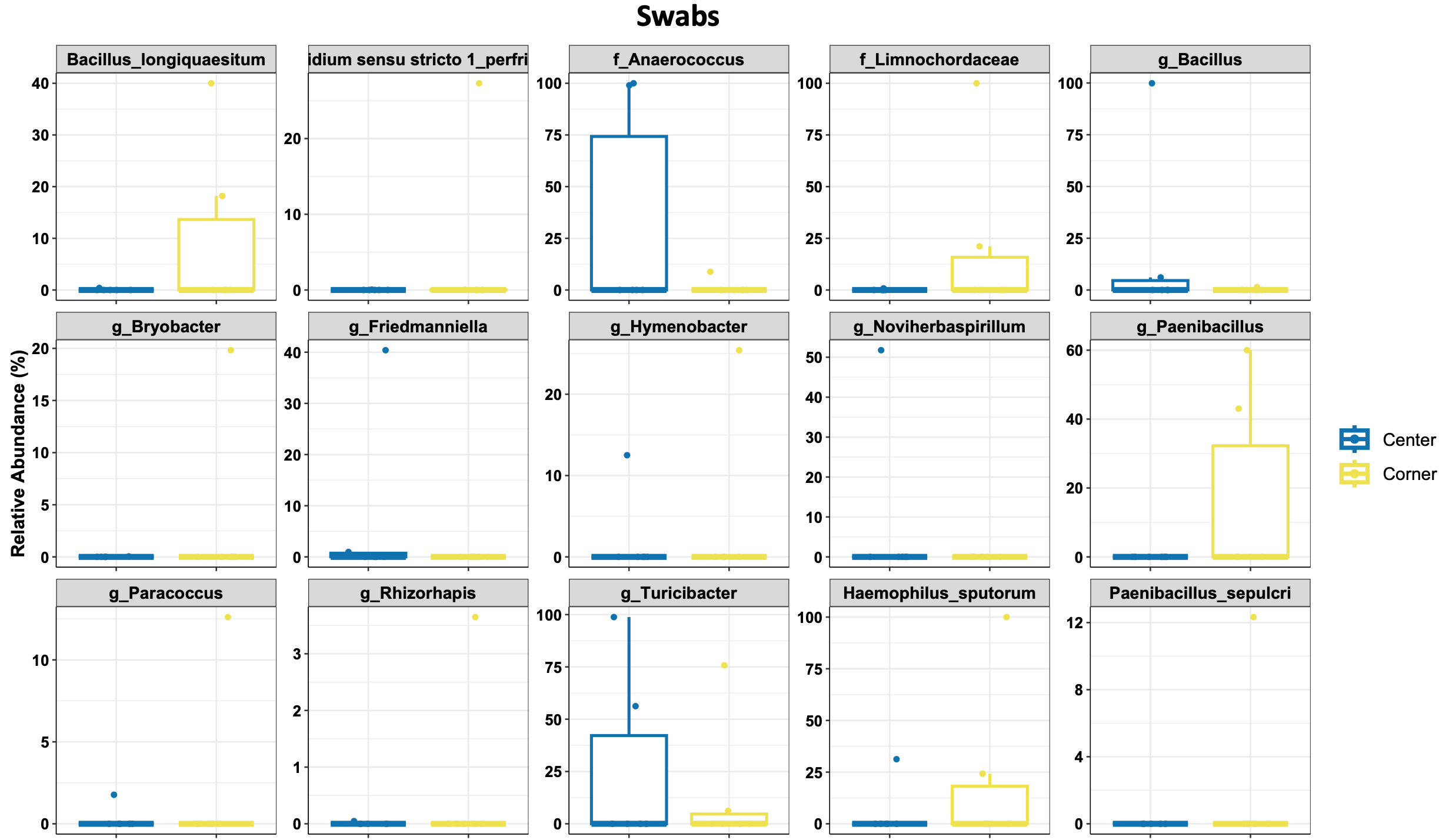


FIGURE S3 Top 15 taxa identified in the swabs. Significant differential abundant changes were identified in the corners and center. The center of the artwork had an abundance of *Anaerococcus* and *Turibacter* sp. The corners of the artwork had an abundance of environmental-associated taxa, *Bacillus longiquaesitum, Limnochordaceae,* and *Paenicacillus* sp. Host-associated bacteria, *Haemophilus sputorum*, was also abundant in the corners of the artwork.


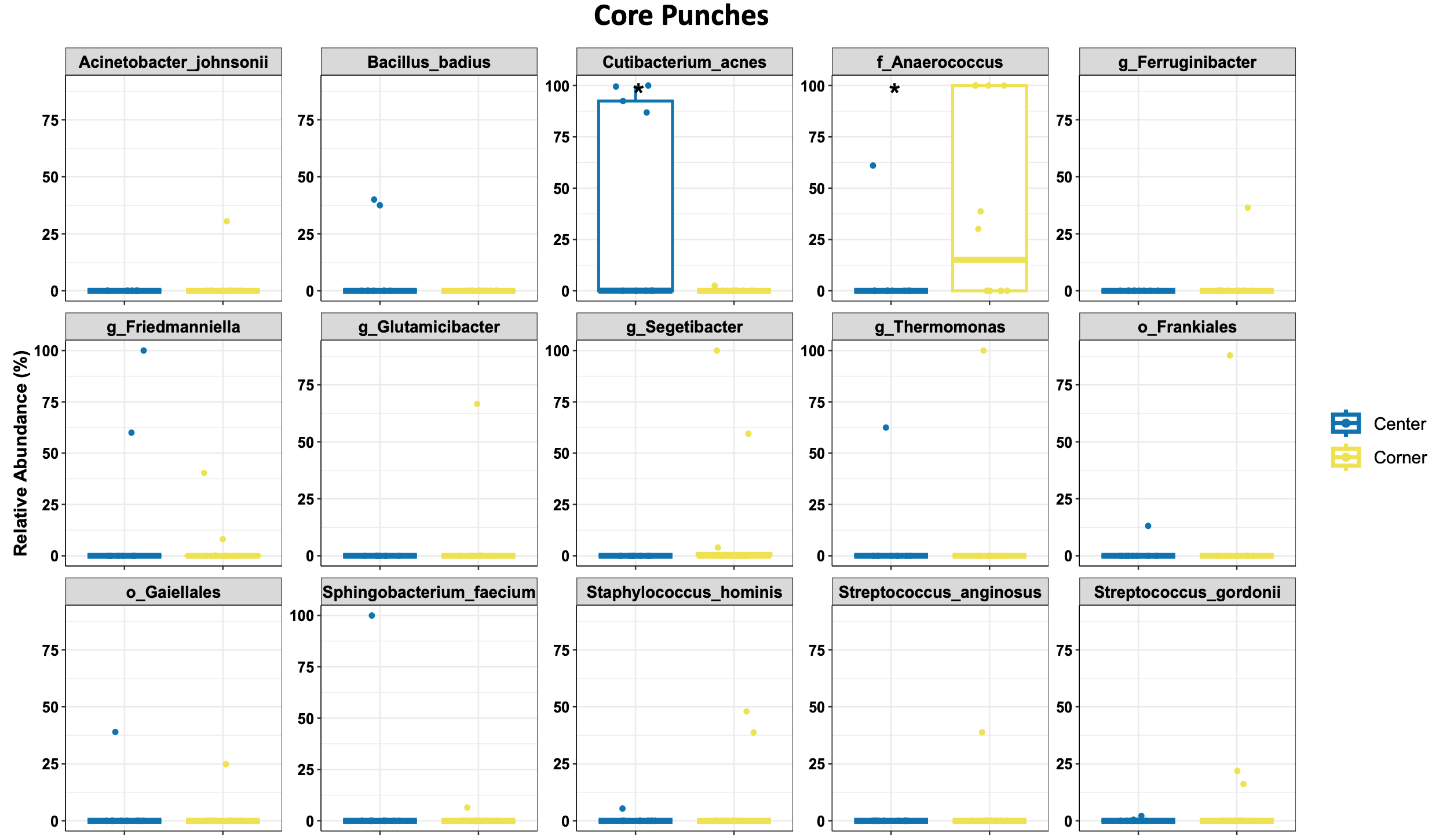


FIGURE S4 Top 15 taxa identified in the core punches. Significant differential abundant changes were identified in the corners and center. Commensal skin bacterium *Cutibacterium acnes* was significantly (p-value <0.05) detected in the center of the artwork. Host-commensal bacteria (commonly isolated from skin, human vagina, nasal cavity, oral cavity and feces), *Anaerococcus* sp., was significantly (p-value <0.05) detected in the corner of the artwork.

**
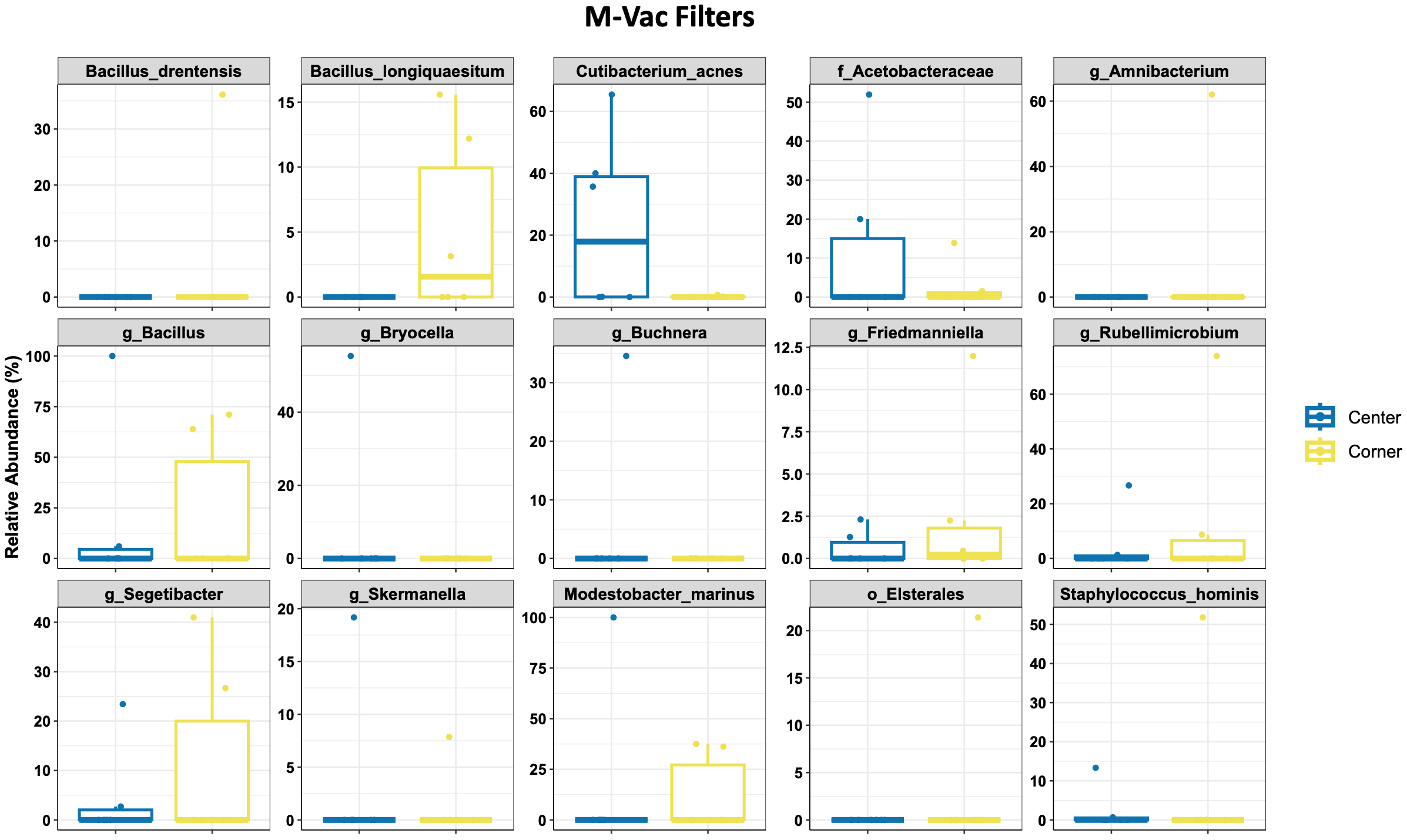
**

FIGURE S5 Top 15 taxa identified in the M-Vac filters. Differential abundant taxa were identified in the corners and center. For instance, *Cutibacterium acnes* (commensal skin bacteria) and Acetobacteraceae (family of strictly anaerobic bacteria that oxidizes ethanol to acetic acid) were abundant in the center. Abundant environmental-associated taxa identified in the corners of the artwork were *Bacillus longiquaesitum, Segetibacter* and *Modestobacter marinus*.

**
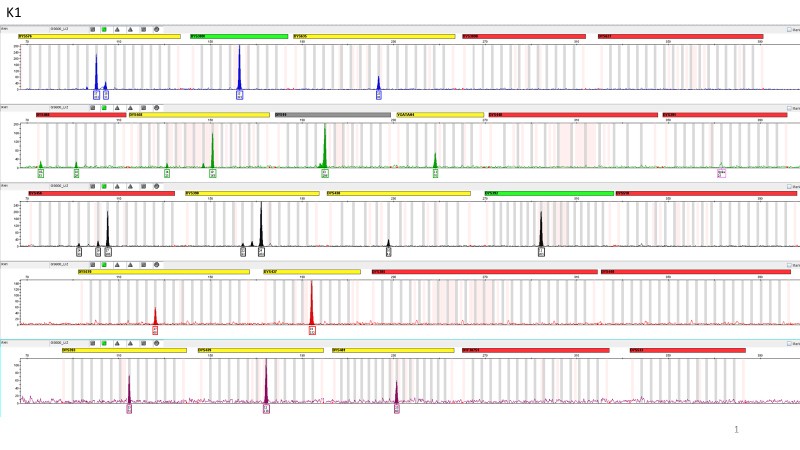
**

**
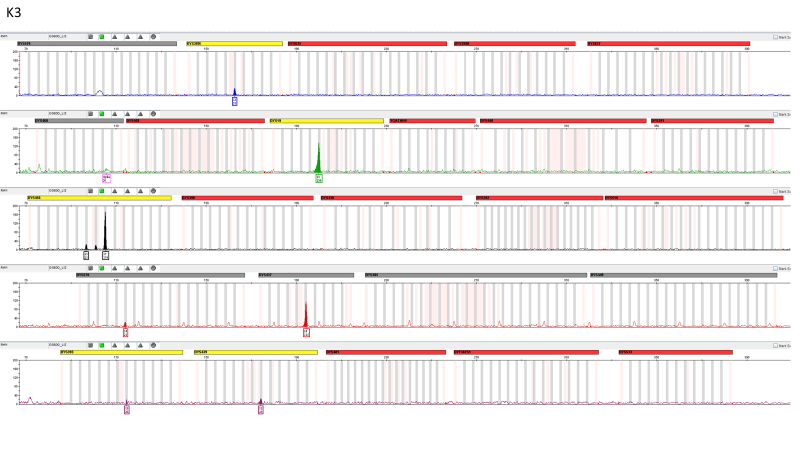
**

**
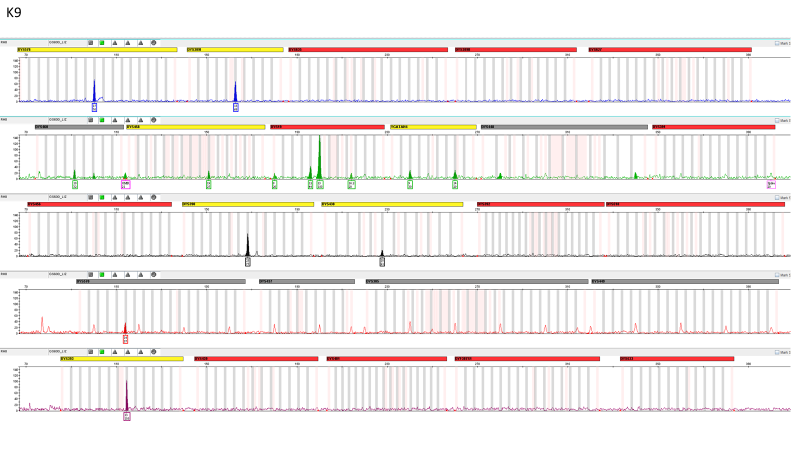
**

FIGURE S6 Top Yfiler Plus results for K1, K3, and K9. Y-STR results were obtained from each artwork, but none produced a full single-source profile. The Y-STRs for K1 resulted in a major peak at 14 Y-STRs with some low-level peaks observed at four of these Y-STRs suggesting a mixture of three or more males; the larger Y-STRs (300 bp) did not amplify. The Y-STRs for K3 resulted in a major peak at three Y-STRs with some low-level peaks observed at five of the Y-STRs suggesting a mixture of two or more males. The Y-STRs for K9 resulted in low-level peaks at 11 Y-STRs with a mixture of two or more males. Given the age of these artworks and their extensive handling over time, the presence of mixed human DNA results was anticipated.
